# Change in Patient Comfort Using Mobile Phones Following the Use of an App to Monitor Tuberculosis Treatment Adherence: Longitudinal Study

**DOI:** 10.2196/11638

**Published:** 2019-02-01

**Authors:** Diana Do, Richard S Garfein, Jazmine Cuevas-Mota, Kelly Collins, Lin Liu

**Affiliations:** 1 San Diego State University San Diego, CA United States; 2 University of California San Diego School of Medicine La Jolla, CA United States

**Keywords:** mHealth, medication adherence monitoring, mobile phone, video technology, tuberculosis

## Abstract

**Background:**

As mHealth apps proliferate, it is necessary for patients to feel capable and comfortable using devices that run them. However, limited research is available on changes in comfort level before and after the use of an mHealth app.

**Objective:**

The objective of this study was to determine whether patients with tuberculosis who used an mHealth app called Video Directly Observed Therapy (VDOT) to monitor their antituberculosis treatment became more comfortable using mobile phones after the intervention and to identify factors associated with change in comfort.

**Methods:**

We analyzed data from a longitudinal study assessing the feasibility and acceptability of the VDOT app among patients receiving antituberculosis treatment from public health departments in San Diego, San Francisco, and New York City. Comfort levels on six domains of mobile phone use (making phone calls, taking pictures, recording videos, text messaging, internet and email use on the phone) were measured on a 10-point scale (1=very uncomfortable; 10=very comfortable) at the start and end of treatment using VDOT via telephone interviews. The main outcomes were change in comfort level on each domain (recoded as binary measures) and an overall change score (sum of individual measures). Linear and logistic regression analyses were performed to assess whether sociodemographics, risk factors, and VDOT perceptions were associated with change of comfort measures.

**Results:**

Among 120 participants with complete data, mean age was 39.8 years (SD 14.8, range 18-87 years), 46.7% (56/120) were female, and 76.7% (92/120) were foreign born. The combined comfort level at baseline was high overall (mean 48.8, SD 14.2, interquartile range 43.0-60.0) and the mean comfort score increased by 1.92 points at follow-up (*P*=.07). Statistically significant increases in comfort on individual domains included taking pictures (*P*=.02) and recording videos (*P*=.002). Females were more likely to have increased comfort in using the internet on the phone compared to males (odds ratio [OR] 3.03, 95% CI 1.08-8.52, *P*=.04). Participants who worked less hours per week were more likely to have increased comfort recording videos although this did not meet statistical significance (OR 1.03, 95% CI 1.00-1.05, *P*=.06).

**Conclusions:**

Findings suggest that, despite a high level of comfort using mobile phones at baseline, experience using the VDOT app was associated with increased comfort using mobile phone features. Additional research involving participants with lower baseline mobile phone experience is needed. An implication of these findings is that as patients begin to use mHealth apps for one health condition, they could acquire skills and confidence to more quickly adapt to using mHealth apps for other conditions.

## Introduction

Mobile phone apps play an increasingly important role in health care as evidence supporting these interventions accumulates [1]. These mHealth (mobile health) interventions have been applied to monitor postoperative care [2,3], diabetes self-management [4,5], smoking cessation [6,7], care and prevention of HIV and other sexually transmitted diseases [8], and more. As these interventions begin to spread throughout health care, there has been a growing interest in the response to these apps, including topics such as acceptability and feasibility [2], satisfaction by patients and providers [5], and efficiency [4]. However, there is limited research measuring users’ comfort in operating mobile phone functions and whether comfort increases as a result of engagement with these apps.

The World Health Organization and Centers of Disease Control recommend the use of directly observed therapy (DOT) for monitoring treatment of both tuberculosis and sometimes latent tuberculosis infection. DOT is a case management system developed for monitoring antituberculosis treatment [9]. Patients with tuberculosis have to travel to a health care facility or a trained DOT worker travels to the patient’s home to observe them taking their medication. DOT improves adherence because it requires close supervision, but it has some barriers as well [10]. One example of a barrier to DOT is that it requires both a patient’s and DOT worker’s presence; both schedules may interfere with time and place of when the medication is taken. These and other barriers prompted the development of an mHealth intervention called Video Directly Observed Therapy (VDOT). VDOT was developed as an alternative to DOT to help reduce the barriers of DOT and increase adherence [11]. The VDOT process allows patients to record themselves taking the medication and send the date- and time-stamped video to a DOT worker who watches the video and documents each dose taken. These recorded videos allow patients the flexibility of choosing the best time and place to take their medication. Patients receive daily medication reminders by short message service (SMS) text message, and may use the phone to text, email, or call their provider throughout treatment.

A study was conducted from August 2013 through July 2014 to assess the feasibility and acceptability of VDOT for monitoring tuberculosis treatment adherence in three US health departments (San Diego, San Francisco, and New York); patient comfort using mobile phones was also measured during baseline and follow-up interviews [11]. In this paper, we assess the change in comfort using mobile phone features and apps and look at factors associated with change. Understanding patient’s comfort and use of mHealth apps will assist in the design and development of other health-based mobile phone apps.

## Methods

### Participant Recruitment

This analysis used data from a three-city study of VDOT among patients with active tuberculosis. Patients 18 years or older undergoing treatment for active tuberculosis in San Diego, San Francisco, and New York City Health Departments were invited to participate in the study by health department staff. Research staff conducted phone interviews before patients started to use the VDOT app (baseline) and after their treatment or study participation ended (follow-up). The interviews included questions on sociodemographics, risk behaviors, VDOT perceptions, and comfort using mobile phone apps and phone features. Participants were provided phones with the VDOT app installed and were told that they could use the phones for anything related to their tuberculosis treatment: making calls, text messaging, emailing, taking pictures, and accessing the internet. The study was approved by the University of California San Diego Human Research Protection Program. All participants provided written informed consent.

### Outcomes

The main outcome for this analysis was change in comfort using mobile phone features between the baseline and follow-up interviews. Comfort with phone features was stratified into six categories: making phone calls, taking pictures, recording videos, text messaging, internet browsing, and using email. Participants were asked, “On a scale of 1 to 10, how comfortable are you using a cell phone to (category)? 1=very uncomfortable and 10=very comfortable.” This scale was developed by the authors and was first used in our study; therefore, we assessed internal reliability using robust alpha coefficients [12]. The estimated alpha was .93 (95% CI .87-.96), which showed excellent reliability. These questions were asked during both the baseline and follow-up interviews. Change in comfort was calculated by subtracting the baseline scores from the follow-up scores. “Not applicable” and “refuse to answer” were potential response options and were treated as missing for this analysis. Overall change in comfort was calculated by summing the six individual technology comfort scores (range 1-10) to produce a cumulative score for baseline and follow-up (range 6-60).

### Data Analysis

The overall change in technology comfort and the change in six separate technology comfort variables were summarized by descriptive statistics (mean, median, quartiles, standard deviation) and assessed by Wilcoxon signed rank test. Univariate tests (Spearman correlation coefficient, Wilcoxon rank sum test, and Kruskal-Wallis test) and simple linear regression analysis were performed to assess the bivariate association between overall comfort change score and each potential covariate (sociodemographics, site, risk behaviors, VDOT perceptions, and baseline comfort score). For individual item comfort change measures, we analyzed them as binary outcomes (improved vs not improved) since normal assumption of residuals for a linear regression model was not satisfied; a change score greater than zero was defined as an improvement. Univariate tests (Wilcoxon rank sum test and Fisher exact test) and simple logistic regression analysis were performed to assess bivariate associations between each individual item change and baseline covariates. Results from the univariate test and regression analysis were consistent; therefore, estimated coefficients, odds ratios (ORs), and *P* values from regression analyses were reported. Variables that were significant with *P*<.10 in the simple linear/logistic regression were considered for inclusion in the multivariable regression analysis. Backward elimination was used to remove nonsignificant variables from the model and the variable with the largest *P* value was removed from the model at each step, until only variables with *P*<.10 remained in the final model. A normal probability plot was used to assess the normality assumption of linear model residuals and influential observations were assessed by residual and Cook’s distance. Sensitivity analyses were performed by excluding the influential observations from the analysis and comparing it to the original model. Since the results remained robust, we report regression analysis results from the original model. A *P*<.05 was interpreted as statistically significant. All data analyses were performed using R (version 3.3.0) and SAS 14 software (Cary, NC, USA).

## Results

### Participant Characteristics

Overall, 151 participants were enrolled in the parent study, of which 126 completed the follow-up interview. Since participants could refuse to answer any question in the interviews, this analysis includes only those who provided responses to at least three comfort questions at both visits, leaving 120 participants for analysis. The mean age was 39.8 years (SD 14.8, range 18-87), 46.7% (56/120) were female, 47.5% (57/120) were Asian, 26.7% (32/120) were Hispanic, and 12.5% (15/120) were African American. Overall, 76.7% (92/120) were foreign born, 55.4% (62/112) reported an annual income of more than US $10,000/year, 79.0% (94/119) had health insurance, and 47.0% (55/117) had education beyond high school. Behavior characteristics showed that 27.7% (33/119) consumed alcohol at least once a month and 40.0% (48/120) had ever smoked cigarettes. Before starting VDOT, 72.5% (87/120) of participants owned a mobile phone and 80.8% (97/120) of participants preferred communicating with their health care provider through text, phone call, or email (text message: 11.7%, 14/120; phone call: 63.3%, 76/120; 14/120; email: 5.8%, 7/120). The mean duration of daily VDOT use was 5.46 months (SD 3.25, interquartile range [IQR] 3.09-7.47).

Overall, mean baseline comfort scores for the six individual domains of mobile phone use were high (range 7.43-9.02; Table 1). The average comfort scores were lowest for email use on phone and highest for making phone calls at both baseline and follow-up. The average change in comfort score was highest for recording videos (mean 0.79, SD 3.04) and lowest for making phone calls (mean 0.11, SD 2.03) and email uses (mean 0.11, SD 2.72); however, the changes were only statistically significant for taking pictures (*P=*.02) and recording video (*P=*.002). Although not statistically significant, an increase was also observed for the combined overall comfort score (*P=*.07).

**Table 1:** Self-reported comfort score using mobile phone features before and after using an mHealth app to monitor antituberculosis treatment adherence among patients in San Diego, San Francisco, and New York City (N=120).

Comfort variables		Baseline	Follow-up	Change	Improvement (change score>0)^a^, n (%)	*P* value^b^
**Making phone calls**					25 (23.6)	.30
	Median (IQR^c^)	10 (9, 10)	10 (9, 10)	0 (0, 0)		
	Mean (SD)	9.02 (1.95)	9.34 (1.52)	0.11 (2.03)		
**Taking pictures**					39 (32.8)	.02
	Median (IQR)	10 (7, 10)	10 (8, 10)	0 (0, 1.5)		
	Mean (SD)	8.21 (2.65)	8.76 (2.29)	0.55 (3.24)		
**Recording videos**					39 (34.2)	.002
	Median (IQR)	9 (6, 10)	10 (8, 10)	0 (0, 1)		
	Mean (SD)	7.69 (3.08)	8.48 (2.38)	0.79 (3.04)		
**Text messaging**					30 (25.9)	.26
	Median (IQR)	10 (8, 10)	10 (8, 10)	0 (0, 1)		
	Mean (SD)	8.23 (2.87)	8.52 (2.55)	0.27 (2.55)		
**Internet use**					28 (26.2)	.58
	Median (IQR)	10 (7, 10)	10 (7, 10)	0 (0, 1)		
	Mean (SD)	7.90 (3.15)	7.95 (3.07)	0.19 (2.64)		
**Email use**					24 (22.9)	.87
	Median (IQR)	9 (5.5, 10)	9 (6, 10)	0 (–1, 0)		
	Mean (SD)	7.43 (3.28)	7.47 (3.13)	0.11 (2.72)		
**Overall comfort**					N/A	.07
	Median (IQR)	54 (43, 60)	56 (50, 60)	0 (–2, 6)		
	Mean (SD)	48.8 (14.2)	51.3 (12.2)	1.92 (11.1)		

^a^Improvement indicates the number and percentage of participants whose change scores were greater than zero for each individual comfort variable.

^b^*P* values are from Wilcoxon signed rank tests.

^c^IQR: interquartile range.

### Combined Overall Comfort

From simple linear regression analysis (Table 2), we found that older age was associated with an increase in overall comfort score change (*P*<.001). Those without a mobile phone at baseline (*P=*.01) and those who had lower comfort at baseline (*P*<.001) were more likely to have increased comfort at follow-up. Using multivariable linear regression, we found only the baseline overall score stayed significant with a higher overall baseline score (ie, more comfortable at baseline) associated with a smaller increase at follow-up (beta=–0.45, *P*<.001).

### Comfort in Six Individual Comfort Domains

To identify factors independently associated with change in comfort level for each of the six individual phone functions, we performed simple (Table 2) and multivariable (Table 3) logistic regression analyses. Variables significantly associated with comfort changes on individual items in the simple logistic regression analysis included age, country of birth, education, hours worked per week, previous mobile phone ownership, ever recording VDOT videos while away from home, and baseline comfort score (all *P*’s<.05).

Variables significantly associated with comfort change for each individual item in simple logistic regression were assessed in multivariable logistic regression models. Baseline comfort levels were the strongest predictors in the individual item change measures (all *P’* s<.001). After controlling for baseline scores, only female participants (OR 3.03, 95% CI 1.08-8.52, *P=*.04) were more likely to have increased comfort in internet use. Participants with fewer hours worked per week (OR 1.03, 95% CI 1.00-1.05, *P=*.06) were also more likely to have increased comfort in recording video although this did not meet statistical significance.

**Table 2:** Simple linear/logistic regression analysis^a^ of change in comfort using mobile phone functions among Video Directly Observed Therapy (VDOT) study participants (N=120).

Variables			n (%)^b^	Overall change, beta	Individual comfort measure, odds ratio					
					Making phone calls	Taking pictures	Recording videos	Text messaging	Internet use	Email use
**Sociodemographics**										
	Age (years), mean (SD)		39.8 (14.8)	0.24^c^	1.02	1.03^d^	1.04^c^	1.04^c^	1.05^c^	1.04^c^
	Hours/week worked at current job, mean (SD)^e^		25.7 (20.4)	0.05	1.04^c^	1.02^f^	1.03	1.01	1.01	1.02
	**Country of birth**									
		Other (ref^g^)	75 (62.5)							
		United States	28 (23.3)	–2.94	0.39	0.34^d^	0.52	0.43	0.45	1.53
		Mexico	17 (14.2)	–2.62	1.28	0.65	1.21	1.56	0.86	0.93
	**Site**									
		San Diego (ref)	43 (35.8)							
		San Francisco	44 (36.7)	0.54	1.00	1.24	1.73	0.50	0.72	1.03
		New York	33 (27.5)	–1.70	0.63	1.15	1.88	1.31	0.66	1.55
	**Race/ethnicity**									
		Asian (ref)	57 (47.5)							
		Hispanic	32 (26.7)	–0.89	1.00	1.20	2.02	1.18	1.35	1.85
		Other	31 (25.8)	–1.03	0.71	1.26	1.44	1.32	0.90	1.03
	Female sex (ref: male)		56 (46.7)	0.46	0.62	0.95	1.28	0.84	2.15^f^	1.25
	>High school education (ref: <high school)		55 (47.0)	–1.53	0.83	0.52	0.42^d^	0.57	0.56	0.39^f^
	Have health insurance (ref: no)		94 (79.0)	2.82	0.66	1.25	1.07	1.15	2.68	1.45
	Household income (US$) >$10,000/year (ref: <$10,000/year)		62 (55.4)	–2.84	0.45^f^	0.50^f^	0.62	0.91	0.71	0.54
**Behavioral characteristics**										
	Alcohol consumed ≥once/month (ref: <once/month)		33 (27.7)	0.97	0.52	1.02	1.45	0.43	0.91	0.62
	Ever smoked cigarettes (ref: never smoked)		48 (40.0)	–2.92	0.46	0.57	0.99	0.63	0.95	1.04
**VDOT characteristics**										
	Days of practice needed to learn VDOT process, mean (SD)		1.53 (1.51)	–0.76	1.27	1.01	1.02	1.14	0.99	0.88
	Miles to tuberculosis clinic from home, mean (SD)		13.0 (10.1)	–0.12	0.98	0.99	1.00	1.00	1.01	0.97
	Owned a mobile phone (ref: no)		87 (72.5)	–6.22^c^	0.37^f^	0.20^c^	0.33^d^	0.23^c^	0.64	0.49
	Used VDOT phone while away from home-ever (ref: never)		84 (71.2)	–3.43	0.82	0.36^d^	0.76	0.67	0.30^d^	0.54
	Preferred communicating with physician in person (ref: text/call/email)		23 (19.2)	1.39	2.01	1.78	1.12	0.81	0.57	0.81
	Baseline comfort score^h^			–0.45^c^	0.06^c^	0.43^c^	0.57^c^	0.61^c^	0.75^c^	0.74^c^

^a^Overall change score was analyzed as a continuous measure and the beta coefficient from simple linear regression was reported for overall change score; individual item change scores were analyzed as binary measures since the normal assumption of residuals in linear regression was not satisfied when analyzed as continuous measures, odds ratios from simple logistic regression were reported for individual item change scores.

^b^Values represent n (%) unless otherwise indicated.

^c^*P*<.01.

^d^*P*<.05.

^e^Beta and odds ratio are interpreted for per 1 hour less.

^f^*P*<.10.

^g^Ref: Reference.

^h^Mean (SD) for baseline comfort scores were presented in Table 1.

**Table 3:** Multivariable regression model^a^ for associations with change in comfort using mobile phone functions among VDOT participants.

Comfort variable	Variables, odds ratio (95% CI)		
	Baseline comfort score	Hours/week worked at current job (per 1 hour less)	Sex (female vs male)
Making phone calls	0.06 (0.02 to 0.18)^b^	N/A^c^	N/A
Taking pictures	0.43 (0.31 to 0.59)^b^	N/A	N/A
Recording videos	0.58 (0.47 to 0.72)^b^	1.03 (1.00 to 1.05)^d^	N/A
Text messaging	0.61 (0.50 to 0.74)^b^	N/A	N/A
Internet use	0.73 (0.62 to 0.85)^b^	N/A	3.03 (1.08 to 8.52)^e^
Email use	0.74 (0.64 to 0.85)^b^	N/A	N/A
Overall score	–0.45 (–0.56 to –0.33)^f^	N/A	N/A

^a^Overall change score was analyzed as a continuous measure and the beta coefficient from multivariable linear regression was reported; individual item change scores were analyzed as binary measures and the odds ratios from multivariable logistic regression were reported.

^b^*P*<.01.

^c^N/A: not applicable.

^d^*P*<.10.

^e^*P*<.05.

^f^Beta coefficient (95% CI) from linear regression analysis.

## Discussion

The main function of the VDOT app was to record and send videos of each medication ingestion event, and we found a significantly positive change in comfort with using a mobile phone to take videos and photos. We also observed a nonstatistically significant increase in comfort using all other mobile phone functions following the use of the VDOT app for monitoring tuberculosis treatment adherence. Except for baseline comfort, sex, and hours worked, we did not find baseline sociodemographics to affect comfort change. This indicates that the use of VDOT has the potential to increase patient skills with various mobile phone functions across a range of patient characteristics, which should increase their willingness and ability to utilize future mHealth apps.

Other mHealth studies considered the usability and receptiveness of app-based tools using different forms of Likert scales [2,7] or qualitative measures from patient responses [5,6] to find important associations related to app quality and user opinions. These studies assessed a variety of health interventions and most had positive results when investigating topics such as usability [3] and acceptability [2,7]. Unlike previous studies, this study quantified comfort with using mobile phone functions and examined correlates of those changes. These measures of change in comfort help to elucidate how patients perceive the use of an mHealth app and how its use can impact comfort using mobile phones. Finding that comfort increased after using an mHealth app suggests that introducing patients to one mHealth app can increase the likelihood that they will be willing and able to engage with future mHealth interventions. The factors that contribute to comfort using mobile phone features have important relevance to whether patients have a good experience using the different tools and benefit from the intended purpose of the app.

Some study limitations must be considered. All participants in this study were undergoing treatment for tuberculosis; thus, this sample is not representative of all patient populations. Tuberculosis in the United States most commonly occurs among immigrants from high tuberculosis-burden countries; persons who are homeless, inject illegal drugs, or are HIV infected; and people who live or work in hospitals, homeless shelters, correctional facilities, nursing homes, and residential homes for those with HIV [13]. The study took place in three major metropolitan cities, so the results might not reflect those of patients in rural areas. Also, the modest sample size might have limited our ability to detect statistically significant associations. Since most participants owned mobile phones and had high comfort levels at baseline, they had little room to increase comfort, which also limited our ability to detect changes. Future studies involving patients with less mobile phone experience at baseline should be considered. Lastly, the data were all self-reported, which may have introduced information bias.

This study has several strengths. The longitudinal study design supports a causal relationship between the observed change in comfort and use of the VDOT app. Baseline and follow-up questionnaires encompassed a wide range of variables including sociodemographic, behavioral, and treatment perceptions, which allowed us to assess multiple potential correlates of change in comfort. The study assessed comfort using six different functions of a mobile phone rather than mobile phone use in general, which allowed for a more comprehensive investigation of the effect of using an mHealth app on change in comfort using mobile phones. Lastly, this is the first study to measure change in comfort using an mHealth app, which helps to address gaps in existing literature.

This study shows that after using a mobile phone for one mHealth intervention, patients became more comfortable using the device, which suggests that they will more quickly embrace other mHealth interventions (eg, smoking cessation or diabetes management). With expanded use of mHealth apps like VDOT, it is important for patients to be comfortable with using mobile phone functions. Measuring the comfort level that patients have while using mHealth apps can help to determine if these apps are improving patient skills using technology. This will help prepare patients to adopt the use of other forms of mHealth, as well as accessing health information through their mobile phone.

## Abbreviations

DOT: directly observed therapy
OR: odds ratio
VDOT: Video Directly Observed Therapy

## References

[1] Whittaker R (2012). Issues in mHealth: findings from key informant interviews. J Med Internet Res.

[2] Semple JL, Sharpe S, Murnaghan ML, Theodoropoulos J, Metcalfe KA (2015). Using a mobile app for monitoring post-operative quality of recovery of patients at home: a feasibility study. JMIR Mhealth Uhealth.

[3] Scott AR, Alore EA, Naik AD, Berger DH, Suliburk JW (2017). Mixed-methods analysis of factors impacting use of a postoperative mHealth app. JMIR Mhealth Uhealth.

[4] Wu Y, Yao X, Vespasiani G, Nicolucci A, Dong Y, Kwong J, Li L, Sun X, Tian H, Li S (2017). Mobile app-based interventions to support diabetes self-management: a systematic review of randomized controlled trials to identify functions associated with glycemic efficacy. JMIR Mhealth Uhealth.

[5] Holtz BE, Murray KM, Hershey DD, Dunneback JK, Cotten SR, Holmstrom AJ, Vyas A, Kaiser MK, Wood MA (2017). Developing a patient-centered mHealth app: a tool for adolescents with type 1 diabetes and their parents. JMIR Mhealth Uhealth.

[6] McClure JB, Hartzler AL, Catz SL (2016). Design considerations for smoking cessation apps: feedback from nicotine dependence treatment providers and smokers. JMIR Mhealth Uhealth.

[7] Finkelstein J, Cha EM (2016). Using a mobile app to promote smoking cessation in hospitalized patients. JMIR Mhealth Uhealth.

[8] Muessig KE, Pike EC, Legrand S, Hightow-Weidman LB (2013). Mobile phone applications for the care and prevention of HIV and other sexually transmitted diseases: a review. J Med Internet Res.

[9] Centers for Disease Control and Prevention, National Center for HIV/AIDS, Viral Hepatitis, STD, and TB Prevention Division of Tuberculosis Elimination (2015). Core Curriculum on Tuberculosis: What the Clinicians Should Know.

[10] Hopewell PC, Pai M, Maher D, Uplekar M, Raviglione MC (2006). International standards for tuberculosis care. Lancet Infect Dis.

[11] Garfein RS, Collins K, Muñoz F, Moser K, Cerecer-Callu P, Raab F, Rios P, Flick A, Zúñiga ML, Cuevas-Mota J, Liang K, Rangel G, Burgos JL, Rodwell TC, Patrick K (2015). Feasibility of tuberculosis treatment monitoring by video directly observed therapy: a binational pilot study. Int J Tuberc Lung Dis.

[12] Zhang Z, Yuan K (2016). Robust coefficients alpha and omega and confidence intervals with outlying observations and missing data: methods and software. Educ Psychol Meas.

[13] Centers for Disease Control and Prevention (2016). TB Risk Factors.

